# *AtPPRT1*, an E3 Ubiquitin Ligase, Enhances the Thermotolerance in *Arabidopsis*

**DOI:** 10.3390/plants9091074

**Published:** 2020-08-21

**Authors:** Yu Liu, Shuya Xiao, Haoran Sun, Linsen Pei, Yingying Liu, Lu Peng, Xuemeng Gao, Yu Liu, Jianmei Wang

**Affiliations:** Key Laboratory of Bio-Resources and Eco-Environment of Ministry of Education, College of Life Sciences, Sichuan University, Chengdu 610065, China; 2017222040059@stu.scu.edu.cn (Y.L.); 2018222040065@stu.scu.edu.cn (S.X.); 2017141241085@stu.scu.edu.cn (H.S.); 2019106500017@whu.edu.cn (L.P.); 2018322040034@stu.scu.edu.cn (Y.L.); 2019322040033@stu.scu.edu.cn (L.P.); gaoxuemeng@stu.scu.edu.cn (X.G.); 2019222045163@stu.scu.edu.cn (Y.L.)

**Keywords:** *AtPPRT1*, *Arabidopsis thaliana*, thermotolerance, positive regulator

## Abstract

E3 ubiquitin ligase plays a vital role in the ubiquitin-mediated heat-related protein degradation pathway. Herein, we report that the expression of *AtPPRT1*, a C3HC4 zinc-finger ubiquitin E3 ligase gene, was induced by heat stress, and the β-glucuronidase (*GUS*) gene driven by the *AtPPRT1* promoter has shown increased activity after basal and acquired thermotolerance. To further explore the function of *AtPPRT1* in heat stress response (HSR), we used the *atpprt1* mutant and *AtPPRT1*-overexpressing lines (OE2 and OE10) to expose in heat shock. In this study, the *atpprt1* mutant had a lower germination and survival rate than those of Col-0 when suffered from the heat stress, whereas OEs enhanced basal and acquired thermotolerance in *Arabidopsis* seedlings. When compared to Col-0 and OEs, loss-of-function in *AtPPRT1* resulted in lower chlorophyll retention and higher content of reactive oxygen species (ROS) after heat treatment. Moreover, the transcript levels of *AtPPRT1* and several heat-related genes (*AtZAT12*, *AtHSP21* and *AtHSFA7a*) were upregulated to greater extents in OEs and lower extents in *atpprt1* compared to Col-0 after heat treated. Hence, we suggest that *AtPPRT1* may act as a positive role in regulating the high temperature by mediating the degradation of unknown target proteins.

## 1. Introduction

In field production, high temperatures can cause devastating damage to crop yields [1,2]. Therefore, how to improve the heat tolerance of plants is of considerable significance in agricultural production [3]. In the process of plant growth, high temperatures will cause endosperm damage and cell death, thereby reducing the germination rate of seeds [4]. During the seedlings of the plant, heat stress will cause cells to lose water and reduce volume, which makes the plants dwarf, enter reproductive growth in advance and greatly reduce yield [5]. Moreover, at the cellular level, the fluidity of the cell membrane increases with increasing temperatures, which in turn affects the function of related membrane proteins [6]. Inside the cell, temperature affects the activity of various enzymes, which in turn regulates the cell’s metabolic activity. These are the behaviors of plant cells in response to heat stress [7,8]. Plants have evolved different signal pathways in response to heat stress environments. Plants can obtain basic thermotolerance in the exposure of lethal temperature stress [9,10]. They can also get a memory of exposure to sublethal heat stress to acquire thermotolerance and adapt to higher temperature stress [10,11].

Heat stress transcription factor (HSF) and heat-shock protein (HSP) play a key role in heat stress response (HSR). The HSFs transcription factor family has 21 members in *Arabidopsis*, with a conserved DNA binding domain (DBD) at the N-terminus and a region that specifically binds to the heat stress element (HSE) [12]. HSFs are classified into three categories—HSFA, HSFB and HSFC—according to the chain length between the hydrophobic residue and the DBD domain [13]. In HSR, HSFs act as the terminal elements of signal transduction and mediate the expression of HSPs. The expression of HSPs is positively correlated with the thermotolerance of plants [14]. The effect of overexpressing single HSF or HSP gene to improve plant heat resistance is not obvious, indicating that HSF and HSP enhance thermotolerance under synergistic conditions [15]. To date, the molecular mechanism of the heat stress response (HSR) dominated by HSF and HSP needs to be further studied. Previous studies showed that abscisic acid (ABA) could enhance thermotolerance by inducing the expression of *HSFA2c* and *HSPs* in tall fescue and *Arabidopsis* [16], so ABA signaling may be cross-linked with HSR. Moreover, jasmonic acid (JA) and salicylic acid (SA) signaling can improve the basic thermotolerance of *Arabidopsis* [17,18].

The ubiquitin-26S proteasome system (UPS) is an important protein degradation pathway in plants [19]. It responds to various external stimuli to promote protein renewal, thereby regulating the ability of cells to maintain their basic functions [20]. In plants, UPS is an important part of defending against environmental stress, such as drought, extreme temperatures and salinity [21,22]. The function of E3 ligase determines their regulatory role in plant stress response pathways [23], and many E3 ligase family members participate in the regulation of HSR. The BPM-CUL3 E3 ligase degrades DREB2A to negatively regulate HSR in *Arabidopsis* [24]. Overexpression of SUMO E3 ligase SlSIZ1 enhanced thermotolerance by regulating heat-related genes [25].

In our previous studies, we have found that *AtPPRT1* plays a crucial role in response to ABA and salt stress [26,27]. In this study, we further investigated the function of *AtPPRT1* in HSR. The results showed that *AtPPRT1* was induced by heat stress, and *AtPPRT1* enhanced thermotolerance in *Arabidopsis* by inducing the expression of heat-related genes. In addition, *AtPPRT1* also reduced the content of reactive oxygen species (ROS) in *Arabidopsis* seedlings after heat treatment. These results confirmed that *AtPPRT1* plays a positive role in HSR.

## 2. Materials and Methods

### 2.1. Plant Materials and Growth Conditions

The *Arabidopsis thaliana* (Col-0) and the T-DNA insertion mutant *atpprt1* (SALK_005268C) were used in this study. The *AtPPRT1*-overexpressing lines (OE2 and OE10) and *ProPPRT1*::GUS transgenic plants were obtained from our previous study [26]. According to previously reported conditions, the vernalized *Arabidopsis* seedlings were sterilized by 0.5% (*v/v*) bleach solution of NaClO for 15 min. Seeds were sown on MS solid medium (with 1% sucrose and 0.65% agar, pH 5.7) and grown in the growth chamber at 22 °C under a 16-h light/8-h dark photoperiod [28].

### 2.2. Quantitative Real-Time-PCR (qRT-PCR) Analysis

Total RNA of 7-day-old *Arabidopsis* seedlings after heat stress were isolated with RNAiso Plus reagents (Takara, Kyoto, Japan). The cDNA was synthesized by the PrimerScript RT reagent Kit (Takara, Kyoto, Japan) using 1500 ng RNA. The qRT-PCR analyses using the TB GREEN Premix Ex Taq kit (Takara, Kyoto, Japan) were performed on the Applied Biosystems 7500 real-time PCR system. The relative transcription expression levels were calculated by the delta-delta Ct method, and *ACTIN2* acts as an internal reference. Primers of relative genes are shown in Supplementary Table S1 (Supplementary Materials). The accession numbers of genes used in this study are shown in Supplementary Table S2 (Supplementary Materials).

### 2.3. GUS Histochemical Staining

According to the previous report [29], 7-day-old *ProPPRT1::GUS* transgenic seedlings grown on MS solid medium were exposed to heat stress, including basal (45 °C for 3 h) and acquired (37 °C for 2 h, 22 °C for 2 h, 45 °C for 2 h) thermotolerance. After heat treatment, the seedlings were collected and stained at 37 °C for 4 h in the GUS staining solution (Real-Times Company, Detroit, MI, USA). The seedlings decolorized with 70% ethanol were placed under a Leica microscope to observe and take pictures.

### 2.4. Phenotypic Analysis

For germination assays, the seeds from each line were treated with heat stress (37 °C or 45 °C for 3 h) and planted on solid MS medium for 7 days. Subsequently, the germination rates of different genotypes were counted.

For survival assays, 7-day-old seedlings grown on MS solid medium were exposed to heat stress, including basal (45 °C for 3 h) and acquired (37 °C for 2 h, 22 °C for 2 h, 45 °C for 2 h) thermotolerance. After heat treatment, the seedlings were continuously grown in the growth chamber for 2–3 days, and the survival rates of different genotypes were calculated.

For chlorophyll estimation, 50 mg of each genotype seedlings were collected and incubated at 37 °C for 4 h in extraction buffer (H_2_O, acetone, ethanol = 1:5:5). The absorbance of the clear supernatant was measured at 663 and 645 nm as described previously [30]. The total chlorophyll content (mg/L) was calculated using the following equation, total Chl (mg/L) = 20.2 × D_645_ + 8.02 × D_663_.

### 2.5. DAB and H_2_DCFDA Staining

H_2_O_2_ was detected by 3,3′-diaminobenzidine (DAB) staining. According to the previous report [31], 7-day-old different-genotype seedlings grown on MS solid medium were exposed to 45 °C for 3 h, and the seedlings were collected and stained at 22 °C for 5 h in 3,3′-diaminobenzidine (DAB) staining solution (1-mg/mL DAB, adjust to pH 3.0 with HCl). The seedlings decolorized with a decoloring solution (glycerol, acetic acid, ethanol = 1:1:3) were placed under a Leica microscope to observe and take pictures.

The ROS production in the seedlings was detected by using fluorescent dye 2′,7′-dichlorofluorescein diacetate (H_2_DCFDA) (Maokang, Shanghai, China), as described previously [32]. The 7-day-old different-genotype seedlings grown on MS solid medium were exposed to 45 °C for 3 h, and the seedlings were collected and stained at 22 °C for 15 min in 50-μM H_2_DCFDA (dissolved in MES-KCl buffer, MES 10 mM, KCl 50 mM, adjust to pH 5.5 with HCl). The seedlings washed with distilled water were placed under a Leica confocal microscope. The fluorescence in cotyledons and roots of seedlings were detected by using an excitation wavelength of 488 nm.

### 2.6. Measurement of H_2_O_2_ and MDA

The 7-day-old different-genotype seedlings grown on MS solid medium were exposed to 45 °C for 3 h, and the seedlings were detected for hydrogen peroxide (H_2_O_2_) and malondialdehyde (MDA) content analysis by using the H_2_O_2_ assay kit and MDA assay kit (Jiancheng, Nanjing, China).

## 3. Results

### 3.1. Expression Pattern Analysis of AtPPRT1 under Heat Stress

**Figure 1:**
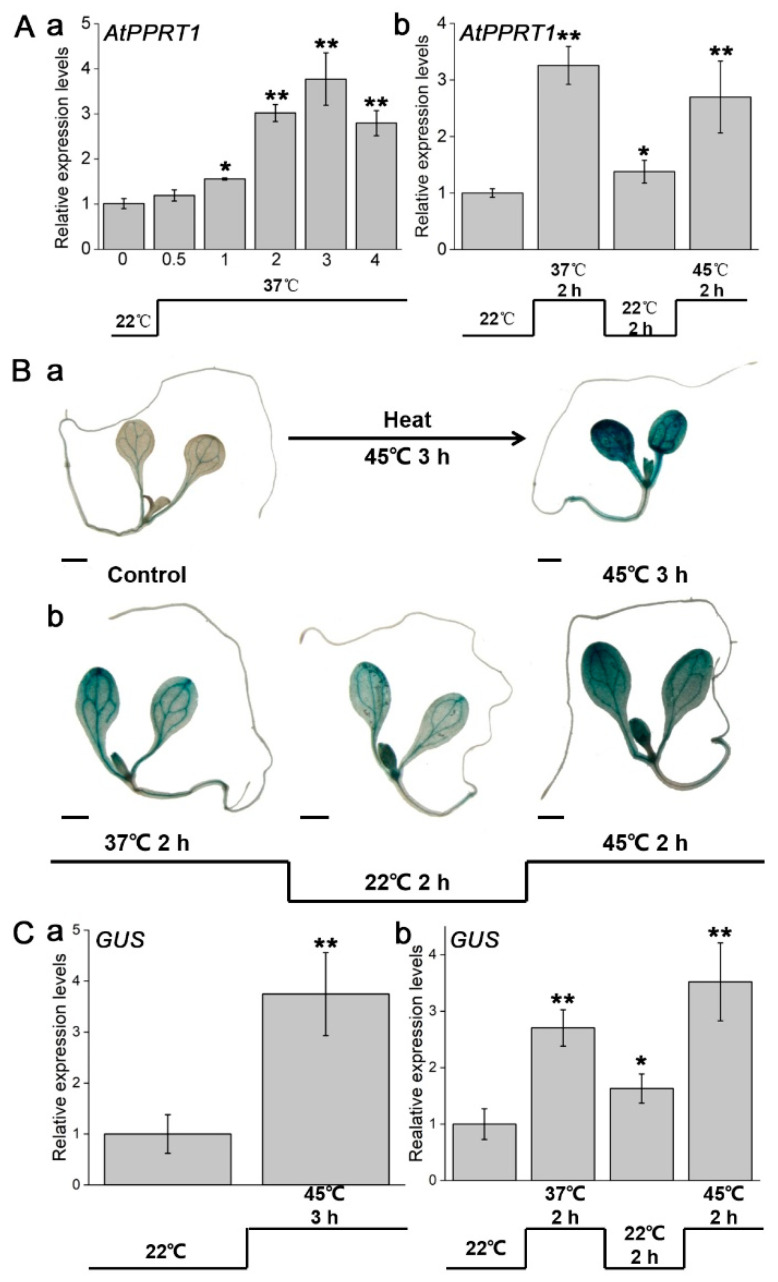
Expression of *AtPPRT1* was induced by heat stress. (**A**) Transcript level of *AtPPRT1* under (**a**) basal and (**b**) acquired heat thermotolerance. Error bars represent ± SD (*n* = 3, * *p* < 0.05, ** *p* < 0.01, *t*-test); (**B**) 7-day-old *ProPPRT1::GUS* transgenic seedlings used for GUS histochemical staining under (**a**) basal and (**b**) acquired heat thermotolerance. Scale bar: 1 mm; (**C**) expression of the *GUS* gene under (**a**) basal and (**b**) acquired heat thermotolerance. Error bars represent ± SD (*n* = 3, * *p* < 0.05, ** *p* < 0.01, *t*-test).

To investigate the expression pattern of *AtPPRT1* in HSR, 7-day-old Col-0 seedlings were exposed to heat stress. Under basal thermotolerance at 37 °C, the expression of *AtPPRT1* continually increased and reached a maximum peak with approximately 4-fold at 3 h (Figure 1(Aa)). The acquired thermotolerance assay showed an increased transcript level of *AtPPRT1* that reached at 3.2-fold after 37 °C treated for 2 h, but subsequently, it decreases to 1.2-fold after 22 °C treated for 2 h, which was still higher than that under normal conditions (approximately 1-fold). Finally, under continuous heat-stress, the transcript level of *AtPPRT1* again increased and reached at 2.7-fold after 45 °C treated for 2 h (Figure 1(Ab)), indicating that *AtPPRT1* is induced by heat stress.

GUS histochemical staining was performed on 7-day-old *AtProPPRT1::GUS* transgenic plants after basal or acquired thermotolerance. Under nonstress conditions, the *GUS* gene driven by the *AtPPRT1* promoter was mainly expressed in veins and hypocotyl vascular bundles of 7-day-old seedlings. Under basal or acquired thermotolerance, GUS activity was significantly upregulated in cotyledons and hypocotyls of *Arabidopsis* seedlings (Figure 1(Ba,b)). In transgenic seedlings, the expression of the *GUS* gene was also induced by heat stress, and the upregulation trend of the *GUS* gene was similar to *AtPPRT1* (Figure 1(Ca,b)). These results indicated that heat stress enhances the activity of *AtPPRT1* promoter, and *AtPPRT1* may play a crucial role in HSR.

### 3.2. AtPPRT1 Enhances Thermotolerance in Arabidopsis

**Figure 2:**
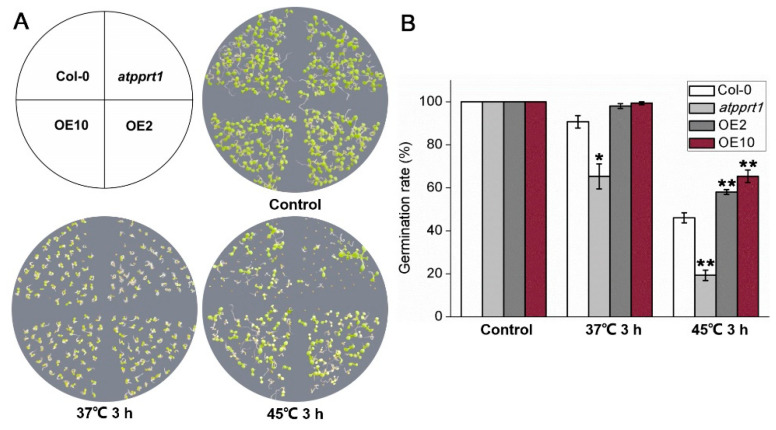
*AtPPRT1* enhances the thermotolerance of *Arabidopsis* seeds during germination. (**A**,**B**) Germination rate of each line after heat stress. Error bars represent ± SD (*n* = 50, * *p* < 0.05, ** *p* < 0.01, *t*-test).

**Figure 3:**
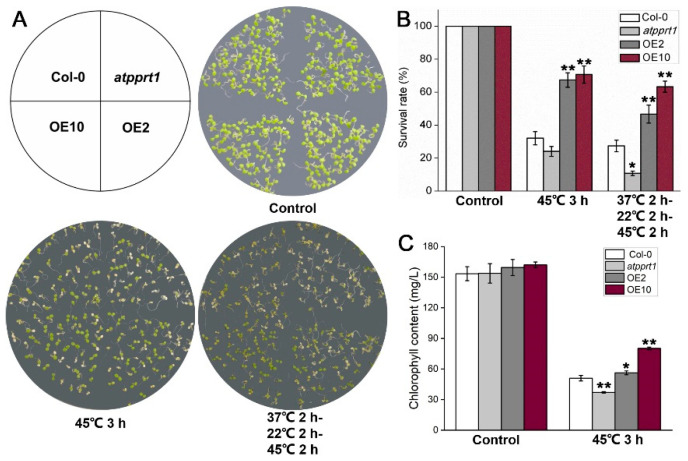
*AtPPRT1* enhances thermotolerance in *Arabidopsis* seedlings. (**A**,**B**) Survival rate of each line after heat stress. Error bars represent ± SD (*n* = 50, * *p* < 0.05, ** *p* < 0.01, *t*-test); (**C**) the chlorophyll content of each line after heat stress. Error bars represent ± SD (*n* = 3, * *p* < 0.05, ** *p* < 0.01, *t*-test).

To further explore whether *AtPPRT1* is involved in HSR, seeds germination rates and seedlings survival rates under heat shock were analyzed in different genotypes. Under control conditions, there was no difference in the germination rate and chlorophyll content of each genotype. After 3 h treatment at 45 °C, the germination rates of OEs were significantly higher than those of Col-0, whereas *atpprt1* showed greatly lower germination rates than Col-0 after 3 h treatment at 37 °C and 45 °C (Figure 2). Similarly, OEs and *atpprt1* mutant showed contrary phenotypes in survival rates of *Arabidopsis* seedlings after basal and acquired thermotolerance (Figure 3A,B). In addition, seedlings of OEs showed increased chlorophyll retention and *atpprt1* showed reduced chlorophyll retention under heat stress with regard to the high temperature treated Col-0 (Figure 3C).

### 3.3. AtPPRT1 Reduced the Accumulation of ROS Caused by Heat Stress in Arabidopsis

**Figure 4:**
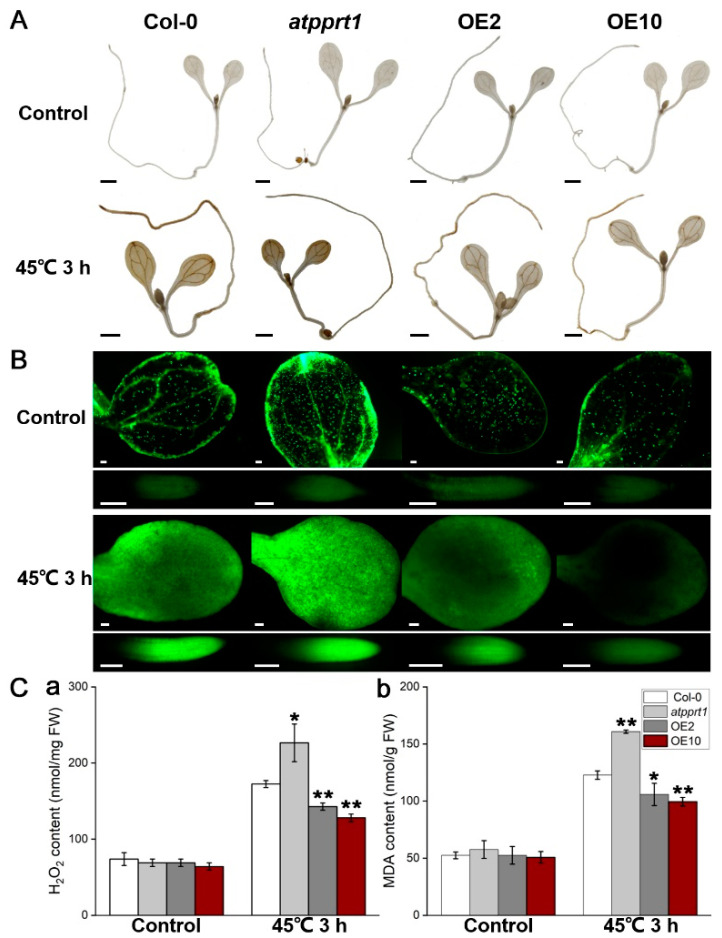
*AtPPRT1* reduces tissue damage caused by heat stress. (**A**) DAB-staining assay of 7-day-old seedlings of Col-0, *atpprt1*, OE2 and OE10 after heat stress. Scale bar: 1 mm; (**B**) detection of ROS in 7-day-old seedlings of different genotypes by using H_2_DCFDA. Seedlings from Col-0, *atpprt1*, OE2 and OE10 were exposed to 45 °C for 3 h. Fluorescence of ROS (green) was imaged in cotyledon and root cells, and the images were collected after 15 min; (**C**) content of (**a**) H_2_O_2_ and (**b**) MDA in Col-0, *atpprt1*, OE2 and OE10 before and after heat stress. Error bars represent ± SD (*n* = 3, * *p* < 0.05, ** *p* < 0.01, *t*-test).

Heat stress leads to the continuous accumulation of reactive oxygen species (ROS) in plants. Previous studies have shown that the interruption of ROS scavenging systems will exacerbate the damage of heat stress to plants, indicating that ROS plays an important role in HSR [33]. The accumulation of ROS caused by heat stress directly reflects the degree of plant oxidation and damage. We detected the accumulation of ROS in different genotypes by DAB and H_2_DCFDA staining. Seven-day-old *Arabidopsis* seedlings were exposed to 45 °C for 3 h, and the seedlings were collected for DAB and H_2_DCFDA staining. The results of DAB staining showed that hydrogen peroxide (H_2_O_2_) was largely accumulated in *atpprt1* and less accumulated in OEs after 45 °C for 3 h, compared with Col-0 (Figure 4A). Moreover, we detected the production of ROS in 7-day-old seedling of Col-0, *atpprt1*, OE2 and OE10 by using the fluorescent dyes H_2_DCFDA. The seedlings were exposed to heat shock (45 °C for 3 h) for studying the physiological response in cotyledon and root of seedlings. The results showed that the fluorescence intensity in the cotyledon and root cells of *atpprt1* was dramatically increased when compared with Col-0 and OEs under heat stress (Figure 4B). To further investigate the degree of ROS-caused membrane oxidative damage in different genotypes, H_2_O_2_ and malondialdehyde (MDA) were quantified under normal and heat stress conditions. The results demonstrated that the H_2_O_2_ and MDA contents in each line showed no significant difference under normal conditions. However, when the seedlings grown on MS solid medium were exposed to 45 °C for 3 h, the H_2_O_2_ and MDA contents of *atpprt1* seedlings were higher than those of Col-0 and OEs, which indicated that loss-of-function in *AtPPRT1* increases plant sensitivity to heat stress (Figure 4Ca,b). These results were consistent with the lower survival rate of *atpprt1* seedlings after heat shock, indicating that the E3 ligase *AtPPRT1* functions as a positive regulator in HSR through affecting the contents of ROS under heat stress.

### 3.4. Altered Expression of AtPPRT1 Leads to Altered Induction of Heat-Related Genes

**Figure 5:**
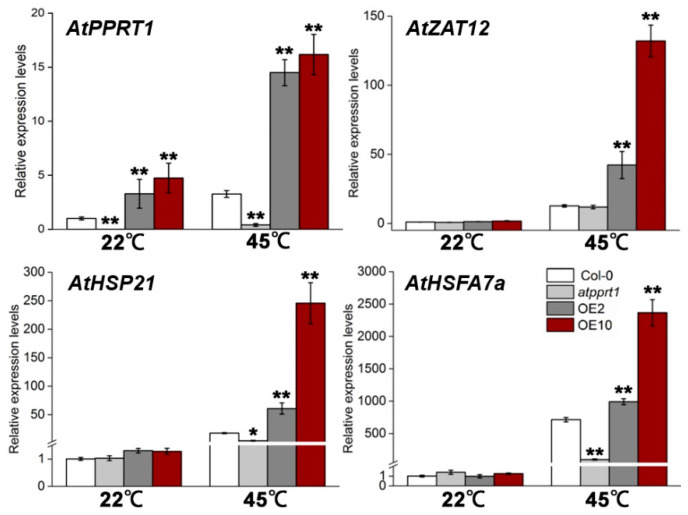
Transcript levels of heat stress-responsive genes were analyzed. Changes of *AtPPRT1*, *AtZAT12, AtHSP21* and *AtHSFA7a* in each line were analyzed by qRT-PCR before and after heat stress treatment. Error bars represent ± SD (*n* = 3, * *p* < 0.05, ** *p* < 0.01, *t*-test).

To further confirm whether the expression of heat-related genes may be affected by *AtPPRT1*, we examined the transcript levels of *AtPPRT1* and several downstream genes in HSR, such as *AtZAT12*, *AtHSP21* and *AtHSFA7a* in different genotypes. The 7-day-old seedling of Col-0, *atpprt1*, OE2 and OE10 were exposed to 45 °C for 3 h and the seedlings were collected for qRT-PCR analysis. Our results showed that the expression of *AtPPRT1* and heat-inducible genes were induced to lower extents in *atpprt1* and greater degrees in OEs compared to Col-0 after heat stress, confirming that *AtPPRT1* acts as a positive regulator in HSR (Figure 5).

## 4. Discussion

In this study, the transcript level of *AtPPRT1* was induced by heat stress for both basal and acquired thermotolerance. The *GUS* gene driven by the *AtPPRT1* promoter was upregulated in transgenic seedlings after heat treatment, indicating that *AtPPRT1* responses to high temperature. After heat stress, the seed germination rates and seedling survival rates of OEs were significantly higher than Col-0, and the transcript levels of *HSP* gene (*AtHSP21*) and *HSF* gene (*AtHSFA7a*) were upregulated to greater extents in OEs and lower extents in *atpprt1* compared to Col-0. HSFA7a is an activator of transcription, which plays a positive role in HSR [34]. HSP21 belongs to small heat shock proteins (sHSP), which is rapidly induced and accumulated under heat stress. Previous studies have shown that the overexpressing of *HSP21* enhanced thermotolerance in *Arabidopsis* and HSP21 also involves a response to temperature-dependent oxidative stress [35,36]. According to previous studies, the C2H2 zinc finger transcription factor ZAT12 is involved in multiple abiotic stresses and could act as crucial roles in reactive oxygen signaling [37]. These results indicated that *AtPPRT1* induces the expression of heat-related genes and maybe enhance basic and acquired thermotolerance by reducing the accumulation of ROS in *Arabidopsis*. Thus, we suggest that *AtPPRT1* plays a positive role in HSR, and we inferred that the degraded target protein downstream of *AtPPRT1* is a negative regulator in the heat-signaling pathway. Moreover, *AtPPRT1* is localized in the mitochondria [26], and the substrate protein may be located in the mitochondria or nucleus. Heat stress may promote the degradation of the substrate protein by *AtPPRT1* in the cytoplasm through nucleocytoplasmic transport—just like KEG degrades ABI5 [38].

Interestingly, in our previous study, it was found that *AtPPRT1* negatively regulates ABA-mediated drought responses in *Arabidopsis*. *AtPPRT1* plays the opposite role in heat and drought stress responses, and we speculate that *AtPPRT1* is involved in the negative crosstalk between heat and ABA stress responses. There have been reports related to this situation. For example, C3HC4–RING finger E3 ligase AtAIRP4 can positively regulate ABA-mediated drought responses in *Arabidopsis*, but overexpression of *AtAIRP4* reduced salt tolerance during the stage of post-germination root growth. *AtAIRP4*-overexpressing lines enhanced sensitive responses to salt and osmotic stresses at seed germination, but the seedlings did not exhibit obvious differences among root length of different genotypes under osmotic stress, so AtAIRP4 may not be involved in osmotic-regulated in the salt signaling pathway. Therefore, the mechanism of AtAIRP4 involved in the reverse regulation of salt and drought stress responses needs further study [39]. In addition, RING-type E3 ubiquitin ligases AtATL78, OsDIRP1 and CaPUB1 have opposite phenotypes in cold and drought stress responses, and they play different functions in cold and drought signaling pathways [40,41,42]. We speculate that *AtPPRT1′*s ubiquitinated substrate maybe two proteins that play opposite roles in heat and drought stress responses. Therefore, it is necessary to identify the targets of *AtPPRT1*, and this will be the next work in the future.

## 5. Conclusions

**Figure 6:**
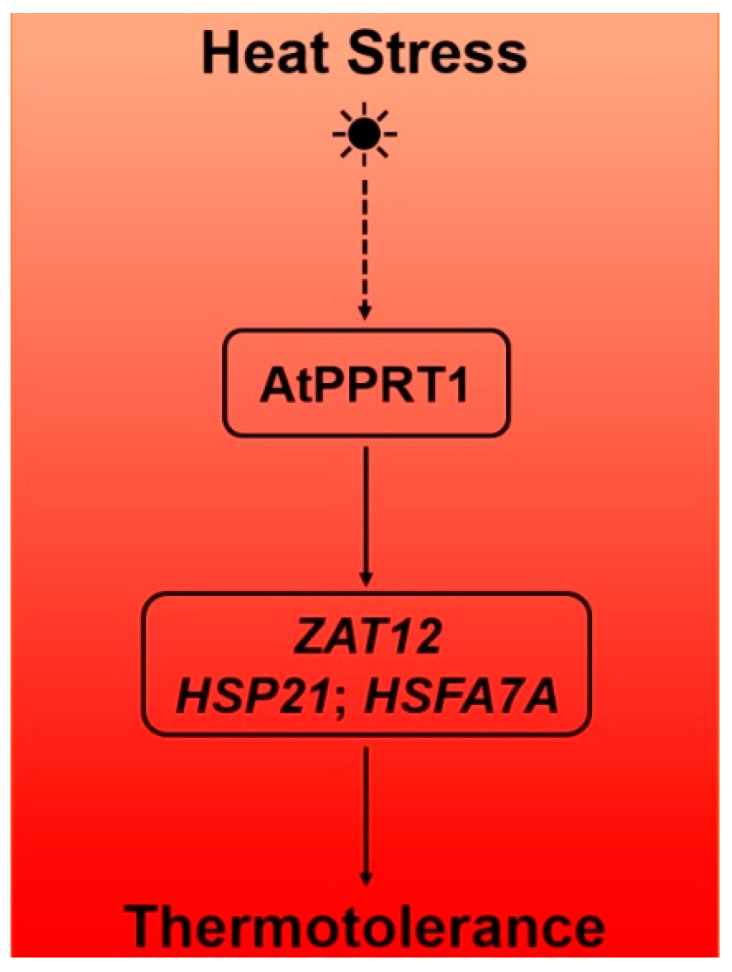
Model illustrating how *AtPPRT1* responds to heat stress in *Arabidopsis*.

We have presented phenotypic and genetic evidence that *AtPPRT1* is induced by heat stress and enhances thermotolerance by reducing tissue damage caused by heat stress. In conclusion, *AtPPRT1* could positively regulate the plant responses to heat stress through induction of the heat shock protein (*AtHSP21*), heat stress transcription factor (*AtHSFA7a*) and heat-related gene (*AtZAT12*) (Figure 6). In addition, the target substrates of *AtPPRT1* and the detailed heat-resistant regulation mechanism needs to be further study.

## Supplementary Materials

The following are available online at https://www.mdpi.com/2223-7747/9/9/1074/s1, Table S1: Primers used in this study, Table S2: The accession numbers of genes used in this study.

## References

[1] Pittelkow C.M., Liang X., Linquist B.A., Van Groenigen K.J., Lee J., Lundy M.E., Van Gestel N., Six J., Venterea R.T., Van Kessel C. (2015). Productivity limits and potentials of the principles of conservation agriculture. Nature.

[2] Haydari M., Zanfardino A., Taleei A., Bushehri A.A.S., Hadian J., Maresca V., Sorbo S., Di Napoli M., Varcamonti M., Basile A. (2018). Effect of heat Stress on yield, monoterpene content and antibacterial activity of essential oils of *mentha x piperita var*. mitcham and *mentha arvensis var*. *piperascens*. Molecules.

[3] Qu A.L., Ding Y.F., Jiang Q., Cheng Z. (2013). Molecular mechanisms of the plant heat stress response. Biochem. Biophys. Res. Commun..

[4] Cheng L., Zou Y., Ding S., Zhang J., Yu X., Cao J., Lu G. (2009). Polyamine accumulation in transgenic tomato enhances the tolerance to high temperature stress. J. Integr. Plant Biol..

[5] Wang Y., Ying J., Kuzma M., Chalifoux M., Sample A., McArthur C., Uchacz T., Sarvas C., Wan J., Dennis D.T. (2005). Molecular tailoring of farnesylation for plant drought tolerance and yield protection. Plant J..

[6] Vigh L., Maresca B., Harwood J.L. (1998). Does the membrane’s physical state control the expression of heat shock and other genes?. Trends Biochem. Sci..

[7] McClung C.R., Davis S.J. (2010). Ambient thermometers in plants: From physiological outputs towards mechanisms of thermal sensing. Curr. Biol..

[8] Suzuki N., Koussevitzky S., Mittler R., Miller G. (2011). ROS and redox signalling in the response of plants to abiotic stress. Plant Cell Environ..

[9] Hasanuzzaman M., Nahar K., Alam M., Roychowdhury R., Fujita M. (2013). Physiological, biochemical, and molecular mechanisms of heat stress tolerance in plants. Int. J. Mol. Sci..

[10] Mittler R., Finka A., Goloubinoff P. (2012). How do plants feel the heat?. Trends Biochem. Sci..

[11] Larkindale J., Hall J.D., Knight M.R., Vierling E. (2005). Heat stress phenotypes of Arabidopsis mutants implicate multiple signaling pathways in the acquisition of thermotolerance. Plant Physiol..

[12] Sakurai H., Enoki Y. (2010). Novel aspects of heat shock factors: DNA recognition, chromatin modulation and gene expression. FEBS J..

[13] Kotak S., Port M., Ganguli A., Bicker F., Von Koskull-Döring P. (2004). Characterization of C-terminal domains of Arabidopsis heat stress transcription factors (Hsfs) and identification of a new signature combination of plant class A Hsfs with AHA and NES motifs essential for activator function and intracellular localization. Plant J..

[14] Nover L., Bharti K., Döring P., Mishra S.K., Ganguli A., Scharf K.-D. (2001). Arabidopsis and the heat stress transcription factor world: How many heat stress transcription factors do we need?. Cell Stress Chaperon..

[15] Kotak S., Larkindale J., Lee U., Von Koskull-Döring P., Vierling E., Scharf K.D. (2007). Complexity of the heat stress response in plants. Curr. Opin. Plant Biol..

[16] Wang X., Zhuang L., Shi Y., Huang B. (2017). Up-regulation of HSFA2c and HSPs by ABA contributing to improved heat tolerance in tall fescue and arabidopsis. Int. J. Mol. Sci..

[17] Clarke S., Cristescu S.M., Miersch O., Harren F.F., Wasternack C., Mur L.A.J. (2009). Jasmonates act with salicylic acid to confer basal thermotolerance in *Arabidopsis thaliana*. New Phytol..

[18] Clarke S., Mur L.A.J., Wood J.E., Scott I.M. (2004). Salicylic acid dependent signaling promotes basal thermotolerance but is not essential for acquired thermotolerance in *Arabidopsis thaliana*. Plant J..

[19] Pickart C.M. (2001). Mechanisms underlying ubiquitination. Ann. Rev. Biochem..

[20] Finley D. (2009). Recognition and processing of ubiquitin-protein conjugates by the proteasome. Ann. Rev. Biochem..

[21] Lyzenga W.J., Stone S.L. (2012). Abiotic stress tolerance mediated by protein ubiquitination. J. Exp. Bot..

[22] Dreher K., Callis J. (2007). Ubiquitin, hormones and biotic stress in plants. Ann. Bot..

[23] Xu F.Q., Xue H.W. (2019). The ubiquitin-proteasome system in plant responses to environments. Plant Cell Environ..

[24] Morimoto K., Ohama N., Kidokoro S., Mizoi J., Takahashi F., Todaka D., Mogami J., Sato H., Qin F., Kim J.-S. (2017). BPM-CUL3 E3 ligase modulates thermotolerance by facilitating negative regulatory domain-mediated degradation of DREB2A in Arabidopsis. Proc. Natl. Acad. Sci. USA.

[25] Zhang S., Wang S., Lv J., Liu Z., Wang Y., Ma N., Meng Q. (2018). SUMO E3 ligase SlSIZ1 facilitates heat tolerance in tomato. Plant Cell. Physiol..

[26] Pei L., Peng L., Wan X., Xiong J., Wan Q., Li X., Yang Y., Wang J. (2019). Expression pattern and function analysis of *AtPPRT1*, a novel negative regulator in ABA and drought stress responses in Arabidopsis. Int. J. Mol. Sci..

[27] Liu Y., Pei L., Xiao S., Peng L., Liu Z., Li X., Yang Y., Wang J. (2020). *AtPPRT1* negatively regulates salt stress response in Arabidopsis seedlings. Plant Signal. Behav..

[28] Wan X., Peng L., Xiong J., Li X., Wang J., Li X., Yang Y. (2019). AtSIBP1, a novel BTB domain-containing Protein, positively regulates salt signaling in Arabidopsis thaliana. Plants.

[29] Liu Y., Peng L., Gao X., Liu Y., Liu Z.-B., Li X., Yang Y., Wang J. (2020). AtPPRT3, a novel E3 ubiquitin ligase, plays a positive role in ABA signaling. Plant Cell Rep..

[30] Pérez-Patricio M., Camas-Anzueto J., Sanchez-Alegria A., Aguilar-Gonzalez A., Gutiérrez-Miceli F., Gomez E.E., Voisin Y., Rios-Rojas C., Grajales-Coutiño R. (2018). Optical method for estimating the chlorophyll contents in plant leaves. Sensors.

[31] Nguyen H.M., Sako K., Matsui A., Suzuki Y., Mostofa M.G., Van Ha C., Tanaka M., Tran L.-S.P., Habu Y., Seki M. (2017). Ethanol enhances high-salinity stress tolerance by detoxifying reactive oxygen species in Arabidopsis thaliana and rice. Front. Plant Sci..

[32] Liu D., Zheng S., Wang X. (2016). Lanthanum regulates the reactive oxygen species in the roots of rice seedlings. Sci. Rep..

[33] Suzuki N., Miller G., Sejima H., Harper J., Mittler R. (2012). Enhanced seed production under prolonged heat stress conditions in Arabidopsis thalianaplants deficient in cytosolic ascorbate peroxidase 2. J. Exp. Bot..

[34] Larkindale J., Vierling E. (2008). Core genome responses involved in acclimation to high temperature. Plant Physiol..

[35] Härndahl U., Hall R.B., Osteryoung K.W., Vierling E., Bornman J.F., Sundby C. (1999). The chloroplast small heat shock protein undergoes oxidation-dependent conformational changes and may protect plants from oxidative stress. Cell Stress Chaperon..

[36] Sedaghatmehr M., Mueller-Roeber B., Balazadeh S. (2016). The plastid metalloprotease FtsH6 and small heat shock protein HSP21 jointly regulate thermomemory in Arabidopsis. Nat. Commun..

[37] Davletova S., Schlauch K., Coutu J., Mittler R. (2005). The zinc-finger protein Zat12 plays a central role in reactive oxygen and abiotic stress signaling in Arabidopsis[w]. Plant Physiol..

[38] Liu H., Stone S.L. (2013). Cytoplasmic degradation of the Arabidopsis transcription factor abscisic acid insensitive 5 is mediated by the RING-type E3 ligase KEEP ON GOING. J. Biol. Chem..

[39] Yang L., Liu Q., Liu Z., Yang H., Wang J., Li X., Yang Y. (2015). ArabidopsisC3HC4-RING finger E3 ubiquitin ligase AtAIRP4 positively regulates stress-responsive abscisic acid signaling. J. Integr. Plant Biol..

[40] Min H.J., Jung Y.J., Kang B.G., Kim W.T. (2016). CaPUB1, a hot pepper U-box E3 ubiquitin ligase, confers enhanced cold stress tolerance and decreased drought stress tolerance in transgenic rice (*Oryza sativa* L.). Mol. Cells.

[41] Cui L.H., Min H.J., Byun M.Y., Oh H.G., Kim W.T. (2018). OsDIRP1, a putative RING E3 ligase, plays an opposite role in drought and cold stress responses as a negative and positive factor, respectively, in rice (*Oryza sativa* L.). Front. Plant Sci..

[42] Kim S.J., Kim W.T. (2013). Suppression of Arabidopsis RING E3 ubiquitin ligase AtATL78 increases tolerance to cold stress and decreases tolerance to drought stress. FEBS Lett..

