# Supplementary material for: AtPPRT1, an E3 Ubiquitin Ligase, Enhances the Thermotolerance in Arabidopsis
Source: Plants (Basel). 2020 Aug 21;9(9):1074. doi: 10.3390/plants9091074 (PMC7569766; doi:10.3390/plants9091074)
Supplement: Supplementary file 1 [file plants-09-01074-s001.pdf]

plants

Article

AtPPRT1, an E3 Ubiquitin Ligase, Enhances the Thermotolerance in *Arabidopsis*

Yu Liu, Shuya Xiao, Haoran Sun, Linsen Pei, Yingying Liu, Lu Peng, Xuemeng Gao, Yu Liu, Jianmei Wang *

Supplementary Materials:

Table S1. Primers used in this study.

ACTIN2-F	ACATCCCACCTACTGGTCTGAAG
ACTIN2-R	GCATCTTGGTATTGCTGGTACTCT
qRT-*AtPPRT1*-F	CATCCTTTTCATGCGCTTAGAG
qRT-*AtPPRT1*-R	AATTCACGAGCTGATGATGTTG
qRT-*GUS*-F	ATACCGAAAGGTTGGGCAGG
qRT-*GUS*-R	TCTTGCCGTTTTCGTCGGTA
qRT-*AtZAT12*-F	GAGTCACAAGAAGCCTAACAACGA
qRT-*AtZAT12*-R	AAGCCACTCTCTTCCCACTGCTA
qRT-*AtHSP21*-F	ATGGCTTCTACACTCTCATTTGCTGCATCGGCTC
qRT-*AtHSP21*-R	AGAGACGTCCATGGTTAAGCGTTGTTGAGGTC
qRT-*AtHSFA7a*-F	AACACTGACCACATCGTATCTT
qRT-*AtHSFA7a*-R	GAGAATCGTGGAGAAAGAATGC

Table S2. The accession numbers of genes used in this study.

Gene Name	Accession Number
AtPPRT1	*AT1G68820*
AtZAT12	*AT5G59820*
AtHSP21	*AT4G27670*
AtHSFA7a	*AT3G51910*
